# Elevated TEFM expression promotes growth and metastasis through activation of ROS/ERK signaling in hepatocellular carcinoma

**DOI:** 10.1038/s41419-021-03618-7

**Published:** 2021-03-26

**Authors:** Lixin Wan, Yang Wang, Zijie Zhang, Jiaxin Wang, Menglan Niu, Yuanyuan Wu, Yating Yang, Yongxia Dang, Shuang Hui, Meng Ni, Bo Wan, Dengke Bao

**Affiliations:** 1 Nanyang central Hospital, School of Pharmacy, Henan University, Kaifeng, Henan 475004 China; 2 Laboratory of Cancer Biomarkers and Liquid Biopsy, School of Pharmacy, Henan University, Kaifeng, Henan 475004 China; 3 College of Animal Sciences and Veterinary Medicine, Henan Agricultural University, Zhengzhou, Henan 450002 China

**Keywords:** Liver cancer, Liver cancer

## Abstract

TEFM (transcription elongation factor of mitochondria) has been identified as a novel nuclear-encoded transcription elongation factor in the transcription of mitochondrial genome. Our bioinformatics analysis of TCGA data revealed an aberrant over-expression of TEFM in hepatocellular carcinoma (HCC). We analyzed its biological effects and clinical significance in this malignancy. TEFM expression was analyzed by quantitative real-time PCR, western blot, and immunohistochemistry analysis in HCC tissues and cell lines. The effects of TEFM on HCC cell growth and metastasis were determined by cell proliferation, colony formation, flow cytometric cell cycle and apoptosis, migration, and invasion assays. TEFM expression was significantly increased in HCC tissues mainly caused by down-regulation of miR-194-5p. Its increased expression is correlated with poor prognosis of HCC patients. TEFM promoted HCC growth and metastasis both in vitro and in vivo by promoting G1–S cell transition, epithelial-to-mesenchymal transition (EMT), and suppressing cell apoptosis. Mechanistically, TEFM exerts its tumor growth and metastasis promoting effects at least partly through increasing ROS production and subsequently by activation of ERK signaling. Our study suggests that TEFM functions as a vital oncogene in promoting growth and metastasis in HCC and may contribute to the targeted therapy of HCC.

## Introduction

Hepatocellular carcinoma (HCC) is one of the most common human cancer type worldwide^1^. Due to the lack of effective method for early detection, most patients are diagnosed at advanced stages with high invasion and metastatic capacities. In addition, despite progress in surgical resection and adjuvant therapy, the prognosis continues to be poor for HCC patients^2^. Therefore, it is critical to identify novel molecular mechanisms driving the metastatic growth of HCC.

Human TEFM (transcription elongation factor of mitochondria) has been identified as a novel nuclear-encoded transcription elongation factor in the transcription of mitochondrial genome, which encodes 13 subunits of the oxidative phosphorylation machinery^3^. Mitochondria play a central role in the regulation of cell metabolism, apoptosis, and reactive oxygen species (ROS) production. It has long been accepted that cancer cells exhibit reprogrammed metabolism, characterized by increased aerobic glycolysis and decreased mitochondrial oxidative phosphorylation, to meet their metabolic demands for development and progression^4^. Additionally, the close link between mitochondrial dysfunction and cancer has been well recognized^5^. However, the roles of mitochondrial DNA transcriptional abnormalities in the contribution to the progression of cancer, especially in HCC, remains largely unclear.

Our bioinformatics analysis of TCGA data revealed an aberrant over-expression of TEFM in HCC, suggesting an oncogenic role for TEFM in HCC. We analyzed its biological effects and clinical significance in this malignancy.

## Materials and methods

### HCC cell lines and tissues sample collection

Human HCC cell lines (SNU-354, SNU-368, SNU-739, HUH-7, HLE, and HLF) and hepatocyte HL-7702 were cultured in DMEM or RPMI-1640 culture medium supplemented with 10% fetal bovine serum (Hyclone) and been recently tested by STR DNA profiling and mycoplasma contamination by our research group. In addition, 239 primary HCC tumor and paired peritumor tissues (30 for qRT-PCR, 209 for immunohistochemistry (IHC) staining) were collected in the First Affiliated Hospital of Zhengzhou University from HCC patients who underwent curative resection. Written informed consent was obtained from all patients. The investigation has been approved by the Ethics Committee of the First Affiliated Hospital of Zhengzhou University in Zhengzhou, China.

### Knockdown and over-expression of target genes

HCC cells in a 6-well plate were transfected with siRNAs targeting human genes of TEFM with lipofectamine 2000 (Invitrogen) per the manufacturer’s instructions. For the construction of shRNA expression vector, siRNA targeting TEFM was cloned into a pSilencer™ 3.1-H1 puro vector (Ambion). For the construction of TEFM expression vector, TEFM was amplified from cDNA and transfected into a pcDNA^TM^3.1(C) vector (Invitrogen, V790-20).

### RNA extraction and qRT-PCR assays

RNA in tissues and cell lines of HCC were extracted with a RNeasy kit (Qiagen, Hiden, Germany) and reversely transcribed into cDNA using a High Capacity cDNA Reverse Transcription Kit (Applied Biosysems, CA, USA). Quantitative real-time PCR (qRT-PCR) was performed on a Corbett 6200 with SYBR Green Dye (Life Technologies). β-actin served as reference gene for normalization. The sequences of forward and reverse primers are provided in Supplementary Table 2.

### Western blotting

HCC tissues and cells were lysed and protein was then electrophoresed on 10% SDS–PAGE gels and transferred onto polyvinylidene fluoride (PVDF) membranes. Nonspecific binding sites on the PVDF membrane were blocked with 5% BSA skimmed milk. After that, the membranes were incubated with primary and secondary antibodies. Primary antibodies used are provided in Supplementary Table 3. The blots were finally imaged with enhanced chemiluminescence system.

### Immunohistochemistry analysis

Paraffin-embedded tissue sections (4 µm thick) were deparaffinized, rehydrated, and treated with hydrogen peroxide for blocking endogenous peroxidase. Antigen retrieval was performed using hot citrate buffer (pH = 6) under pressure. After that, sections were incubated with primary antibodies provided in Supplementary Table 3 for 10 h followed by detection by IHC kit (Invitrogen). Images were analyzed with an Olympus microscope.

### MTS cell viability assay

HCC cells were plated in a 96-well cell culture plate (020096, Xinyou Biotech, Hangzhou, China) at a density of 2 × 10^3^ cells per well. Cell viability was determined by adding 20 ml MTS assay solution (Promega, G3581) according to manufacturer’s instructions. The absorbance at 490 nm was obtained using a Bio-Rad’s microplate reader.

### Apoptosis assays

For the determination of apoptosis in in vitro cell lines, an Annexin V (FITC-conjugated) apoptosis kit (F-6012, US Everbright Inc.) was used per the manufacturer’s instructions. Results were analyzed by flow cytometry (Beckman, Fullerton, CA).

For the determination of apoptosis in tissue, a TUNEL detection kit purchased from Roche biotech (11684795910) was used per the manufacturer’s instructions. The percentage of TUNEL-positive cells was analyzed using a fluorescence Olympus microscope.

### Colony formation assay

HCC cells were seeded in a 6-well plate (1 × 10^3^ cells/well) and cultured for 15 days. The colonies were fixed by paraformaldehyde and then stained using crystal violet solution. The number of colonies were calculated and pictured.

### Cell cycle distribution analysis

Cell cycle distribution was analyzed with a cell cycle kit (F-6012, US Everbright Inc.) following the manufacturer’s instructions. The population of cells in each phase was determined with flow cytometry (Beckman, Fullerton, CA).

### Wound-healing cell migration assay

Cells were plated in a 6-well cell culture plate. When cells were grown to 90–95% confluence, a scratch was made in the middle of the wells. Images were acquired along the cell-free zone with a light Olympus microscope at 0 and 48 h. Relative cell migration was assessed using the ImageJ software.

### Matrigel invasion assay

For invasion assay, 24-well transwell chamber with Matrigel (BD Bioscience) was used following the manufacturer’s instructions. HCC cells were seeded onto the upper chamber wells in serum-free medium and incubated for 48 h. Penetrated cells on the lower membrane were then fixed with 4% paraformaldehyde and stained with 0.1% crystal violet. Stained cells in each chamber were photographed and numbered with a light Olympus microscope.

### Tumor xenograft mouse model

To explore the effect of TEFM on the tumorigenicity of HCC in vivo, 1 × 10^7^ TEFM stable knockdown or control SNU-354 cells were subcutaneously injected into the flanks of 4-week-old male BALB/c nude mice (12 mice were randomized into two groups). Tumor volumes were measured every week for 5 weeks. The mice were sacrificed and tumor nodules were harvested. All animal study procedures were approved by the Institutional Animal Care and Use Committee at the First Affiliated Hospital of Zhengzhou University in Zhengzhou, China.

### Tail vein metastatic assay

To evaluate the effect of TEFM on the metastasis of HCC in vivo, 5 × 10^6^ TEFM stable knockdown or control SNU-354 cells were injected intravenously through the tail vein of 4-week-old male BALB/c nude mice. The mice were sacrificed 6 weeks after cell injection and the tumor nodules formed in the lungs were counted.

### Oxygen consumption rate (OCR) detection

HCC cells with TEFM knockdown or over-expression were plated onto an XF96 plate (1.0 × 10^4^ cells per well) and cultured at 37 °C for 12 h. After that, the oxygen consumption rate (OCR) of each cell group was detected by the XF96 Extracellular Flux Analyzer (Seahorse Bioscience) according to the manufacturer’s protocol.

### Statistical analysis

Experiments were repeated 3 times, where appropriate. The results were expressed as mean ± SEM. SPSS software (17.0 version, Chicago, IL) was used for calculation of statistical significance, and *p*-value < 0.05 was considered as statistically significant. For comparison between two groups, Student’s *t*-test was used. For comparisons among multiple groups, one-way analysis of variance (ANOVA) with Fisher’s least significant difference (LSD) test was used. Correlations between two measured variables were analyzed with Pearson’s correlation coefficient.

## Results

### Up-regulation of TEFM predicts aggressive tumor behavior and poor prognosis of patients with HCC

**Fig. 1:**
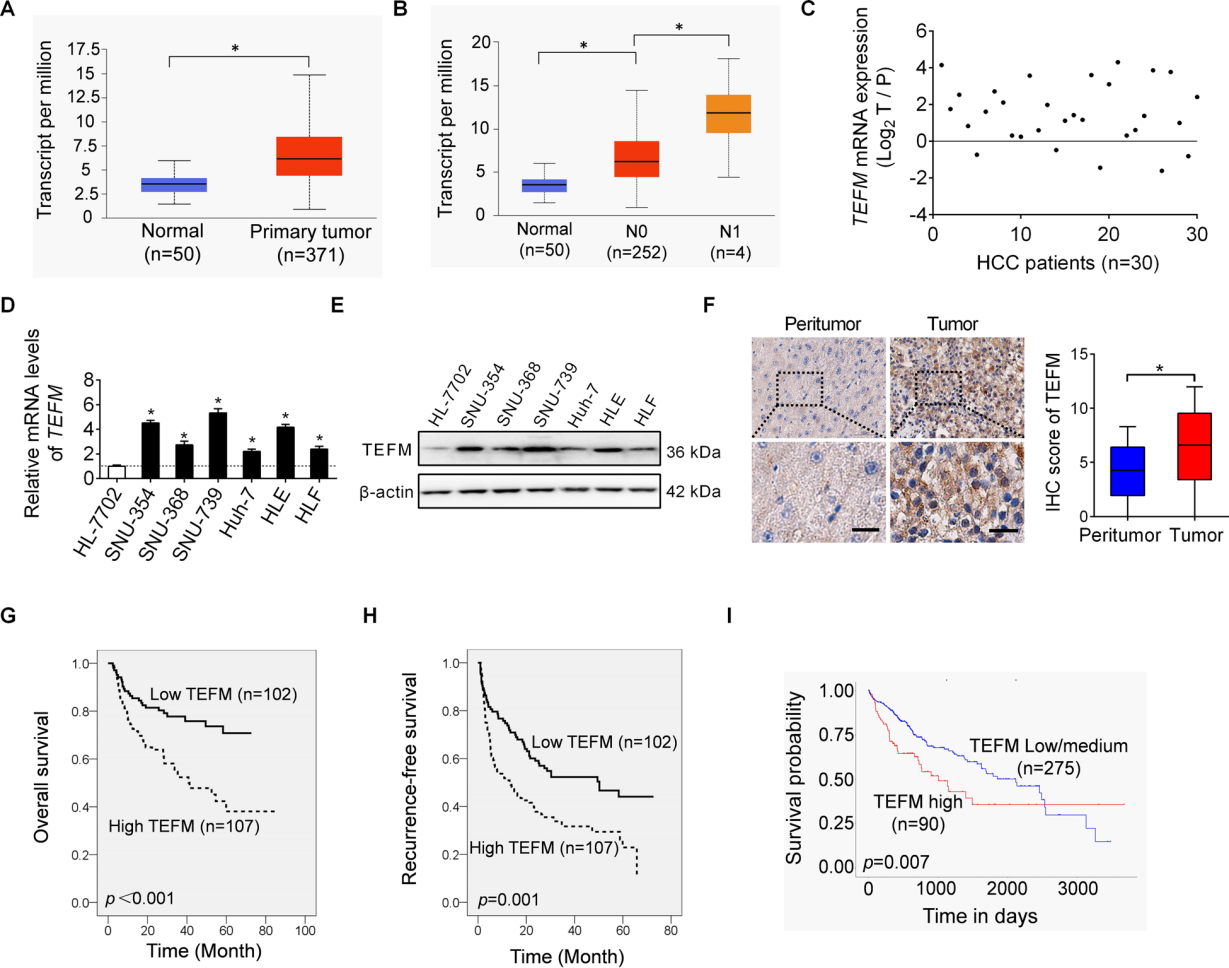
Up-regulation of TEFM predicts aggressive tumor behavior and poor prognosis of patients with HCC. **A**, **B** Bioinformatics analysis for TEFM expression based on the TCGA data. **C** TEFM expression was analyzed by quantitative real-time PCR (qRT-PCR) in paired tumor (T) and peritumor (P) tissues (*n* = 30). **D**, **E** TEFM expression was analyzed by qRT-PCR and western blot in HCC cell lines and normal hepatocyte. **F** Immunohistochemistry (IHC) staining of TEFM in paired tumor and peritumor tissues (*n* = 209). Scale bar, 20 μm. **G**, **H** Prognostic significance of TEFM was analyzed based on IHC data. The median TEFM expression in the 209 HCC patients was chosen as the cut-off. **I** Prognostic significance of TEFM was analyzed using the online web portal UALCAN (http://ualcan.path.uab.edu).

Our bioinformatics analysis using the online web portal UALCAN (http://ualcan.path.uab.edu)^6^ revealed a significant up-regulation of TEFM in tumor tissues of HCC when compared to normal tissues (Fig. 1A). Besides, TEFM expression is increased in node metastasis (N1) HCC than in no node metastasis (N0) HCC (Fig. 1B). The expression of TEFM was further analyzed by qRT-PCR in paired tumor and peritumor tissues from 30 HCC patients. Up-regulation of TEFM was observed in 83% (25/30) tumor tissues compared with their peritumor tissues (Fig. 1C). In line with the results from tissues, TEFM expression was also higher in HCC cell lines as compared with normal hepatocyte (Fig. 1D, E).

To evaluate the clinical significance of TEFM, TEFM expression was analyzed by IHC staining in another 209 paired tumor and peritumor tissues from HCC patients. In keeping with the expression at mRNA, TEFM expression at protein level was also elevated in tumor tissues compared with peritumor tissues (Fig. 1F). In addition, TEFM expression was positively correlated with the diameter of tumors and incidence of portal vein tumor thrombosis (PVTT) (Supplementary Table 1). Moreover, survival analysis showed that patients with high TEFM levels experienced shorter overall survival (OS) and recurrence-free survival (RFS) compared with those have low TEFM levels (Fig. 1G, H), which was further supported by the bioinformatics prognostic significance analysis from the online web portal UALCAN (Fig. 1I) and Kaplan–Meier Plotter^7,8^ (Fig. S1A–1D). These data suggest that TEFM is up-regulated in HCC cells, and its up-regulation predicts a poor prognosis for patients with HCC.

### TEFM knockdown suppressed HCC cell growth by inhibiting G1–S cell cycle transition and inducing cell apoptosis

**Fig. 2:**
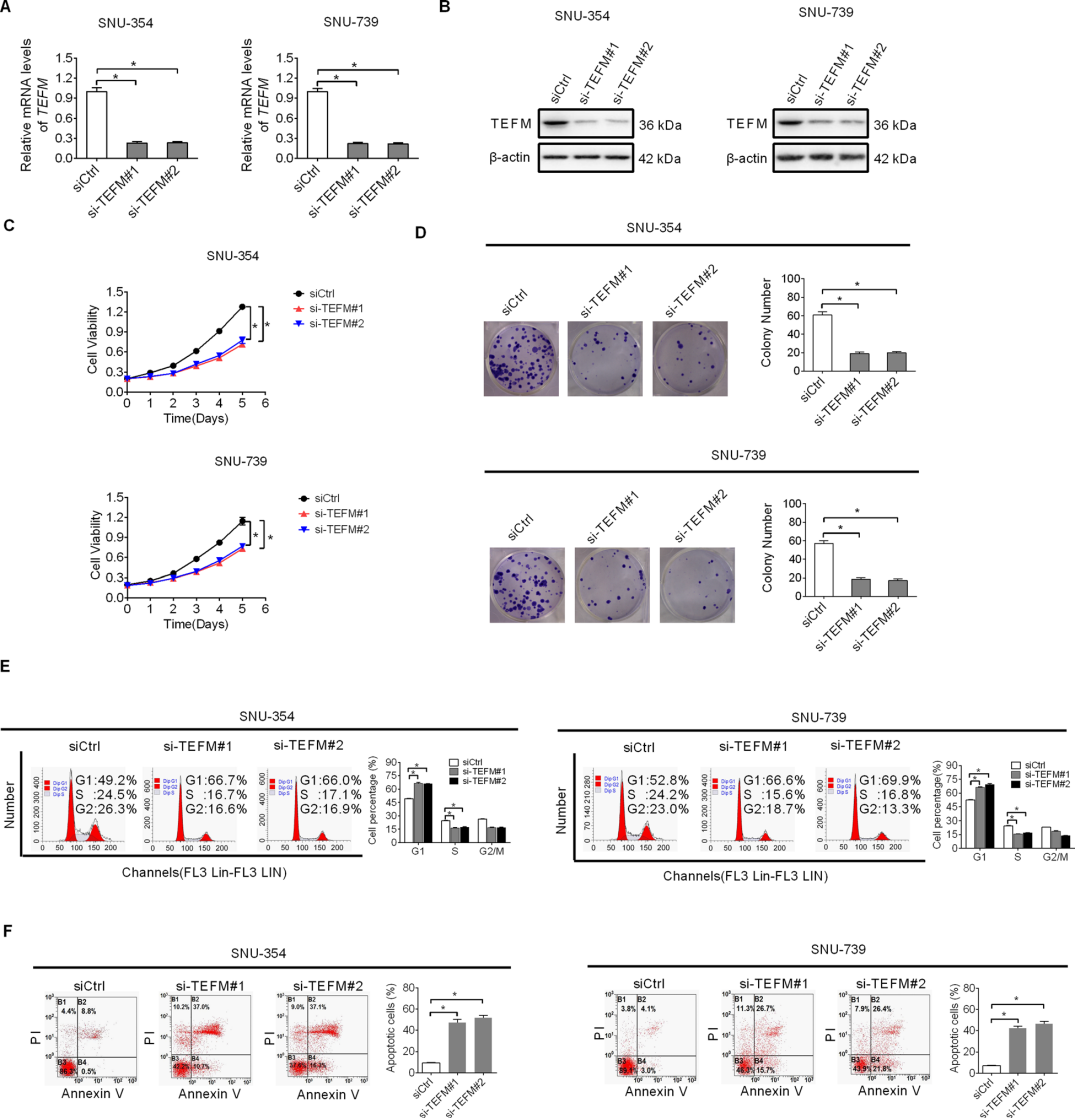
TEFM knockdown suppressed HCC cell growth by inhibiting G1–S cell cycle transition and inducing cell apoptosis. **A**, **B** TEFM expression was analyzed by qRT-PCR and western blot in SNU-354 and SNU-739 cells (siTEFM, siRNA against TEFM; siCtrl, control siRNA). **C**, **D** MTS and colony formation assays were applied for cell growth analysis in SNU-354 and SNU-739 cells. **E** Cell cycle was analyzed by flow cytometry in SNU-354 and SNU-739 cells. **F** Cell apoptosis was analyzed by flow cytometry in SNU-354 and SNU-739 cells.

To elucidate the effect of TEFM on the malignant phenotypes of HCC cells, TEFM expression was knocked-down by RNA interference in SNU-354 and SNU-739 cells, which have relatively high TEFM expression levels (shown in Fig. 1D, E). TEFM knockdown was verified by qRT-PCR and western blot analysis (Fig. 2A, B). MTS and colony formation assays indicated that TEFM knockdown significantly inhibited cell proliferation of SNU-354 and SNU-739 cells (Fig. 2C, D). Because of that, both accelerated cell cycle progression and decreased apoptosis could contribute to increased cell proliferation; the effects of TEFM on cell cycle distribution and apoptosis was thus explored to determine the molecular mechanism by which TEFM promotes HCC cell proliferation. As shown in Fig. 2E, TEFM knockdown resulted in a significant arrest of G1-to-S cell cycle in SNU-354 and SNU-739 cells. Cell apoptosis analysis demonstrated significantly more apoptotic cells in TEFM-knockdown SNU-354 and SNU-739 cells than control cells (Fig. 2F). These results demonstrate that TEFM promotes HCC growth via inducing cell cycle transition from G1 to S and inhibiting cell apoptosis.

### TEFM knockdown suppressed HCC cell invasion and migration through inhibition of epithelial–mesenchymal transition (EMT)

**Fig. 3:**
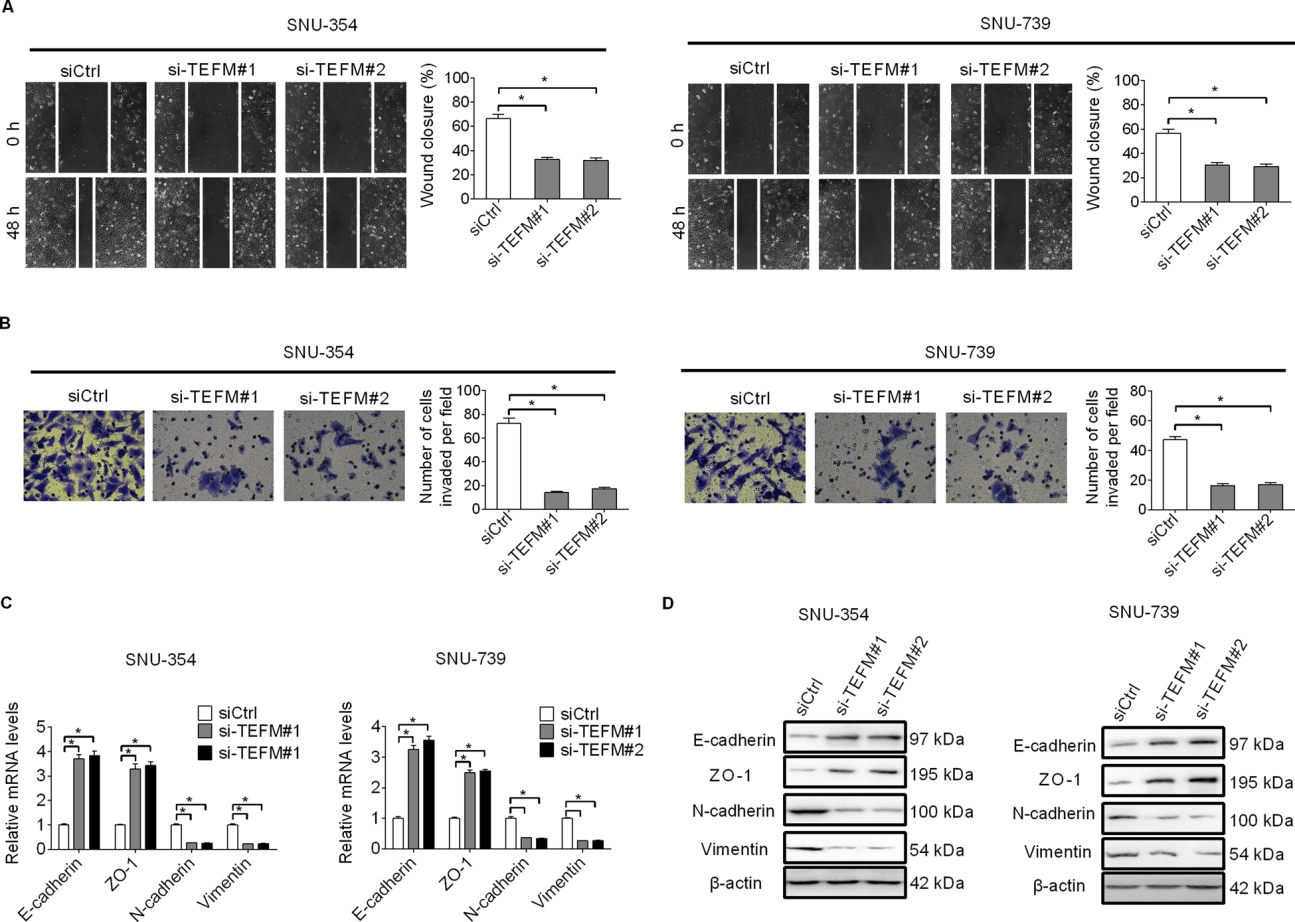
TEFM knockdown suppressed HCC cell invasion and migration through inhibition of epithelial–mesenchymal transition (EMT). **A** Cell migration was analyzed by wound-healing assay in SNU-354 and SNU-739 cells (siTEFM, siRNA against TEFM; siCtrl, control siRNA). **B** Cell invasion was determined by transwell Matrigel invasion assay in SNU-354 and SNU-739 cells after transfection with siTEFM or siCtrl. **C**, **D** Quantitative real-time PCR and western blot analysis for expression levels of EMT markers (E-cadherin, ZO-1, N-cadherin, and Vimentin) in SNU-354 and SNU-739 cells after transfection with siTEFM or siCtrl.

We next performed wound-healing migration and transwell invasion assays to evaluate the effects of TEFM on the metastatic potential of HCC cells. Knockdown of TEFM significantly inhibited the migration and invasion of SNU-354 and SNU-739 cells (Fig. 3A, B). Since epithelial–mesenchymal transition (EMT) plays a critical role in invasion and metastasis of human cancers^9^, the effect of TEFM knockdown on the expressions of EMT markers were analyzed by qRT-PCR and western blot. TEFM knockdown significantly increased the levels of epithelial markers (E-cadherin and ZO-1), while decreased the levels of mesenchymal markers (N-cadherin and Vimentin) in SNU-354 and SNU-739 cells (Fig. 3C, D), suggesting that TEFM may facilitate the migration and invasion of HCC cells through induction of EMT.

### TEFM knockdown suppressed HCC growth and metastasis in vivo

**Fig. 4:**
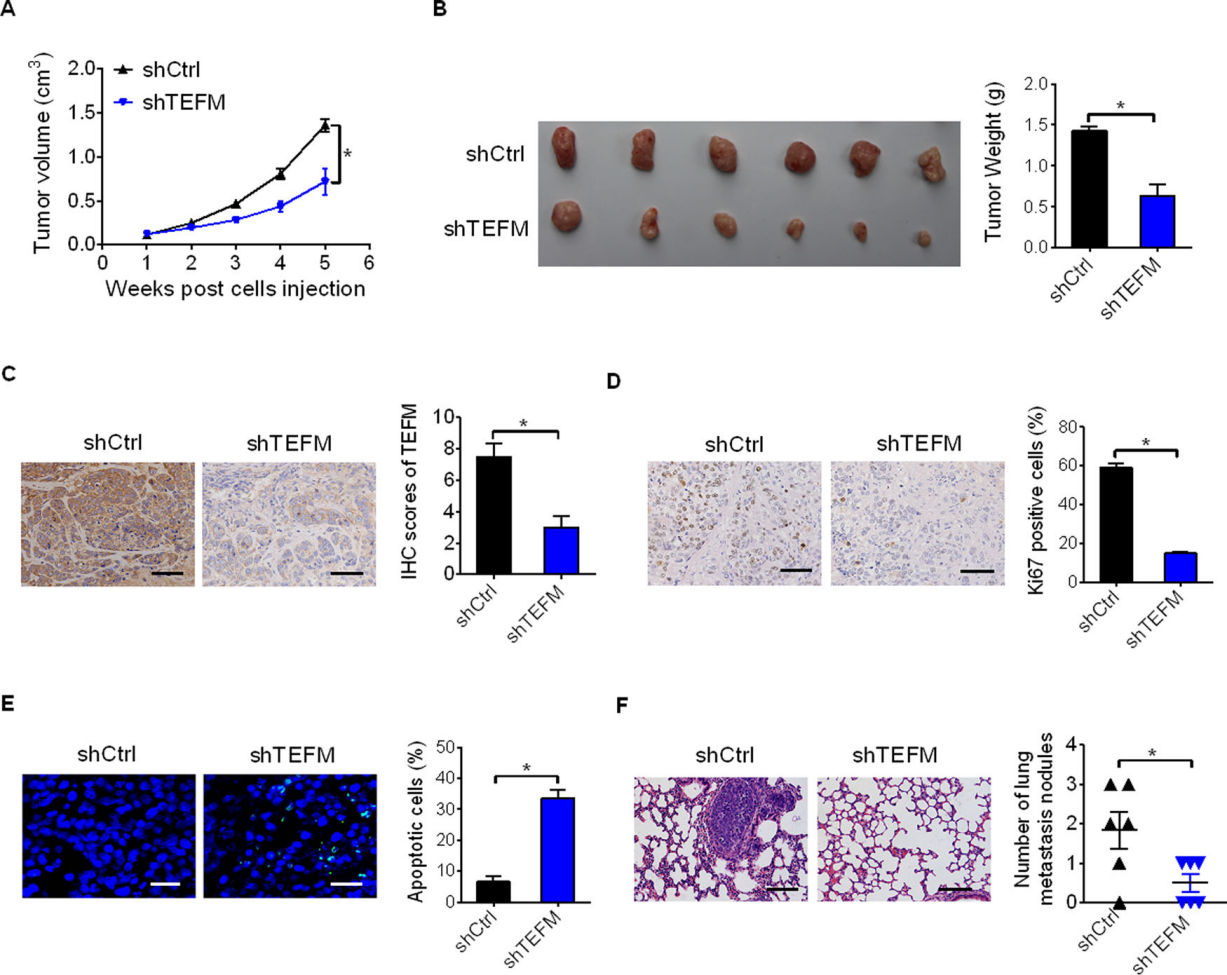
TEFM knockdown suppressed HCC growth and metastasis in vivo. **A** Tumor volumes were measured once a week for 5 weeks. **B** Representative image of gross tumors from the mice. Tumor weight was also measured. **C**, **D** Immunohistochemistry analysis for the expressions of TEFM and Ki-67 in xenograft tumors from the mice. Scale bars, 10 μm. **E** TUNEL staining for cell apoptosis analysis in xenograft tumors from the mice. Scale bars, 5 μm. **F** Incidences of lung metastases was evaluated. Scale bars, 10 μm.

To explore the effect of TEFM on the growth and metastasis of HCC in vivo, TEFM stabley knocked-down (shTEFM) or control (shCtrl) SNU-354 cells (Fig. S2A, 2B) were injected subcutaneously into the flanks of BALB/c nude mice. As shown in Fig. 4A, B, TEFM knockdown dramatically inhibited tumor formation and growth ability of SNU-354 cells in vivo. IHC staining confirmed a significant decrease of TEFM in tumor tissues from shTEFM group than those from shCtrl group (Fig. 4C), implying that the tumor-suppressive effect was exerted by TEFM knockdown. In line with the results from in vitro cell lines, significantly fewer proliferating and more apoptotic cells were observed in tumor tissues from shTEFM group than those from shCtrl group, as evidenced by Ki-67 and TUNEL staining assays (Fig. 4D, E). Moreover, the in vivo tail vein metastasis assay demonstrated significantly fewer metastatic nodules in the lungs of shTEFM group than those of shCtrl group (Fig. 4F).

### Over-expression of TEFM increased HCC cell growth and metastasis

**Fig. 5:**
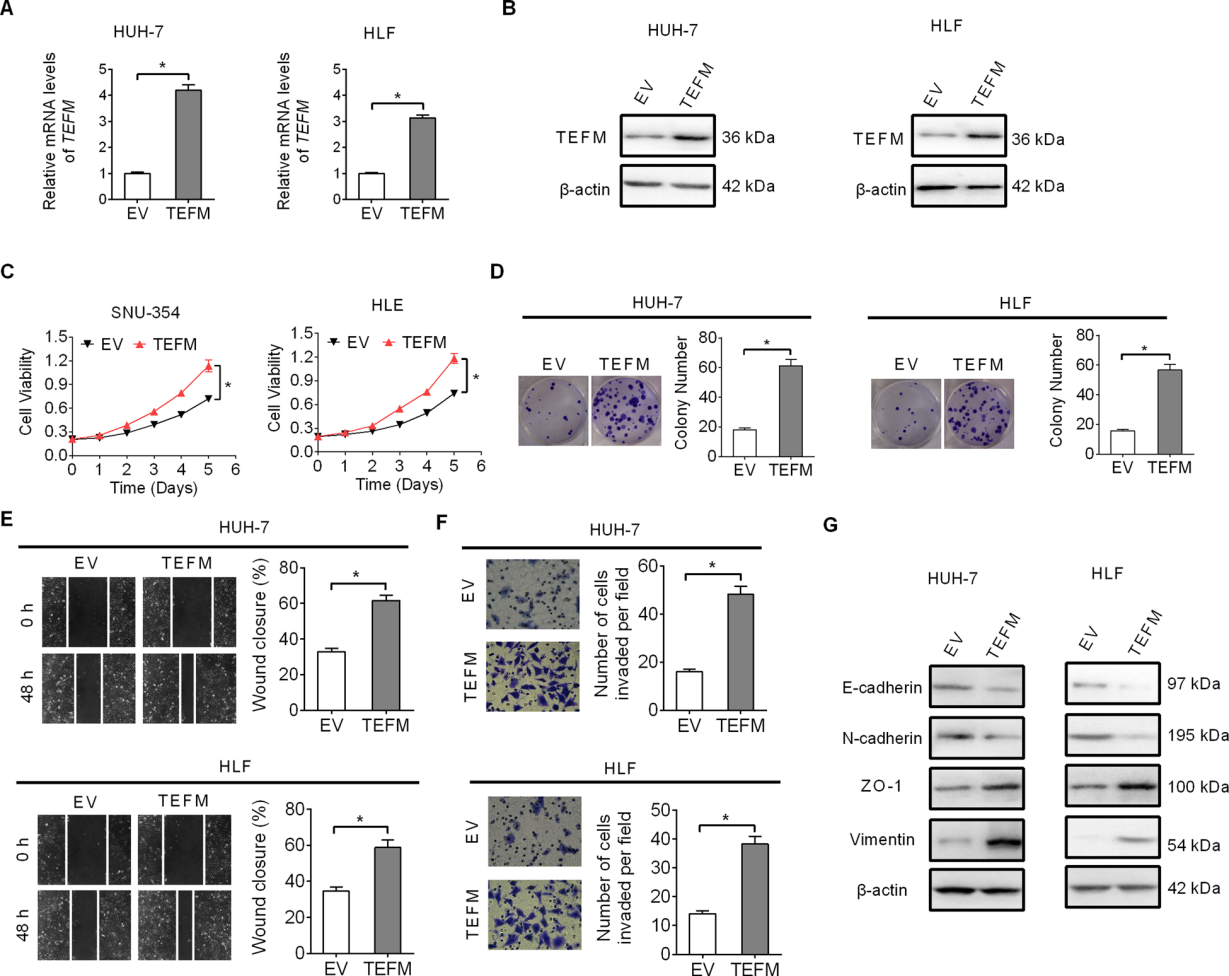
Over-expression of TEFM increased HCC cell growth and metastasis. **A**, **B** TEFM expression was analyzed by qRT-PCR and western blot in HUH-7 and HLF cells (TEFM, TEFM expression vector; EV, empty vector). **C**, **D** Cell growth was analyzed by MTS and colony formation assays. **E**, **F** Cell metastasis was analyzed by wound-healing and Matrigel invasion assays. **G** The expression levels of EMT markers (E-cadherin, ZO-1, N-cadherin, and Vimentin) was analyzed by western blot.

We also over-expressed TEFM to provide further support on the oncogenic functions of TEFM in HUH-7 and HLF cells (Fig. 5A, B), which have relatively low TEFM expression (shown in Fig. 1D, E). Significant increase of cell viability and colony formation was observed in HUH-7 and HLF cells after over-expression of TEFM (Fig. 5C, D). In addition, TEFM over-expression also markedly increased migration and invasion abilities of HUH-7 and HLF cells (Fig. 5E, F). The results were further supported by significantly down-regulated epithelial markers of E-cadherin and ZO-1, while up-regulated mesenchymal markers of N-cadherin and Vimentin upon TEFM over-expression (Fig. 5G), as analyzed by western blot.

### Increased TEFM expression is mainly mediated by down-regulation of miR-194-5p

**Fig. 6:**
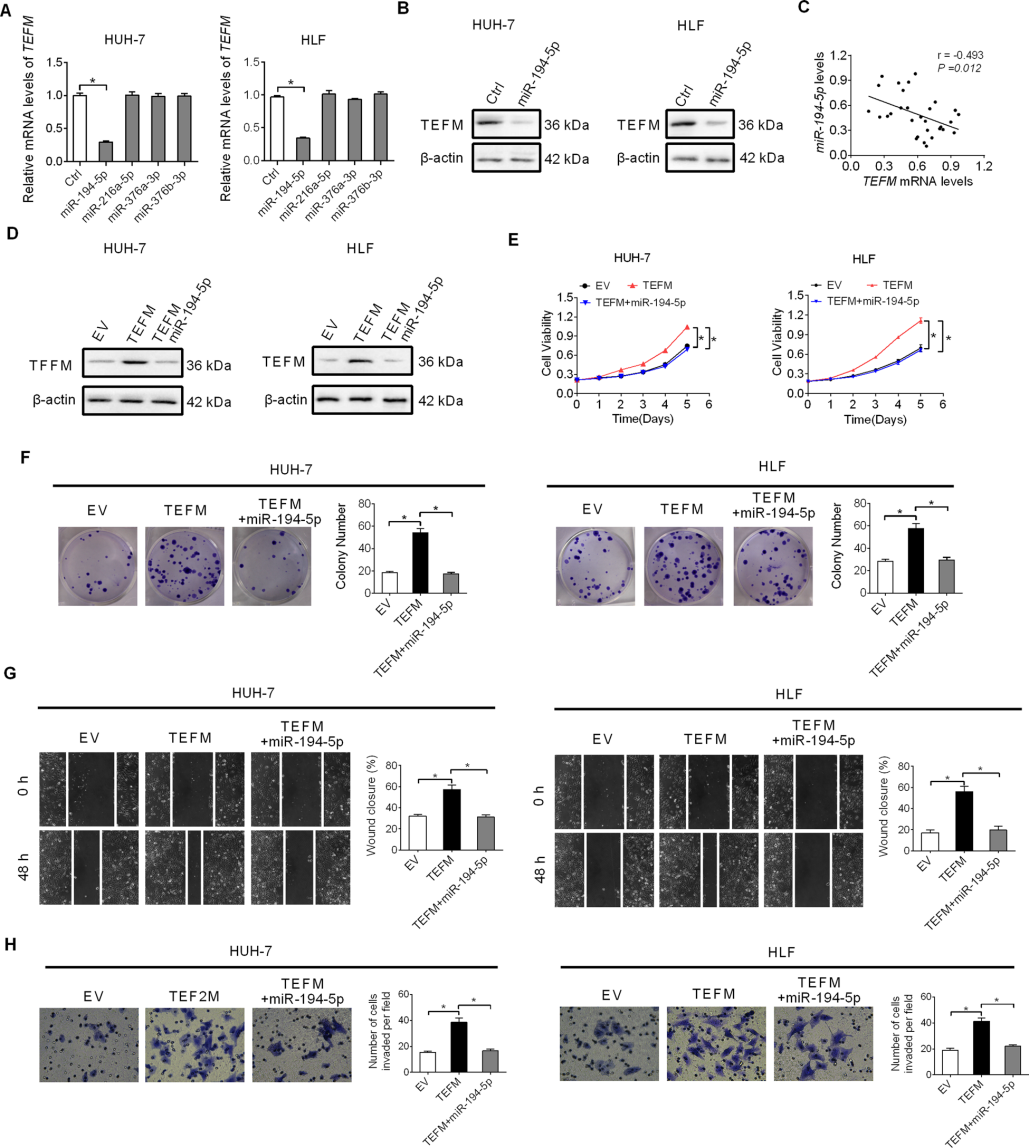
Increased TEFM expression is mainly mediated by down-regulation of miR-194-5p. **A** TEFM expression was analyzed by qRT-PCR in HUH-7 and HLF cells transfected with synthetic precursors of miR-194-5p, miR-216-5p, miR-376a-3p, and miR-376b-3p. **B** TEFM expression were analyzed by western blot in HUH-7 and HLF cells transfected with synthetic miR-194-5p precursor. **C** Correlation between TEFM and miR-194-5p in tumor tissues of HCC (*n* = 30). **D** TEFM expression were analyzed by western blot in HUH-7 and HLF cells transfected with TEFM expression vector and synthetic miR-194-5p precursor. **E**, **F** Cell proliferation was analyzed by MTS and colony formation assays in HUH-7 and HLF cells transfected with TEFM expression vector and synthetic miR-194-5p precursor. **G**, **H** Cell metastasis was analyzed by wound-healing and transwell Matrigel invasion assays in HUH-7 and HLF cells transfected with TEFM expression vector and synthetic miR-194-5p precursor.

MicroRNA is a critical regulator of gene expression at the post-transcriptional level in eukaryotic organisms. To identify potential microRNAs that contribute to the over-expression of TEFM in HCC, the web tool microRNA Data Integration Portal (mirDIP) was used for miRNA target prediction. Among the top four predicted miRNAs targeting TEFM (Fig. S3A), TEFM expression was decreased in HUH-7 and HLF cells only upon transfection of miR-194-5p (Fig. 6A, B). The expression of miR-194-5p was negatively associated with TEFM expression in tumor tissues from 30 HCC patients (Fig. 6C; *r* = −0.493, *p* = 0.012). The results were further supported by the bioinformatics analysis in the ENCORI database^10^ (Fig. S3B). In addition, bioinformatics analysis also showed that miR-194-5p down-regulation predicts poor survival for patients with HCC (Fig. S3C). Furthermore, we found that transfection of miR-194-5p greatly attenuated TEFM-promoted HCC growth and metastasis (Fig. 6D–H).

### TEFM promoted HCC growth and metastasis through activation of ROS/ERK signaling

**Fig. 7:**
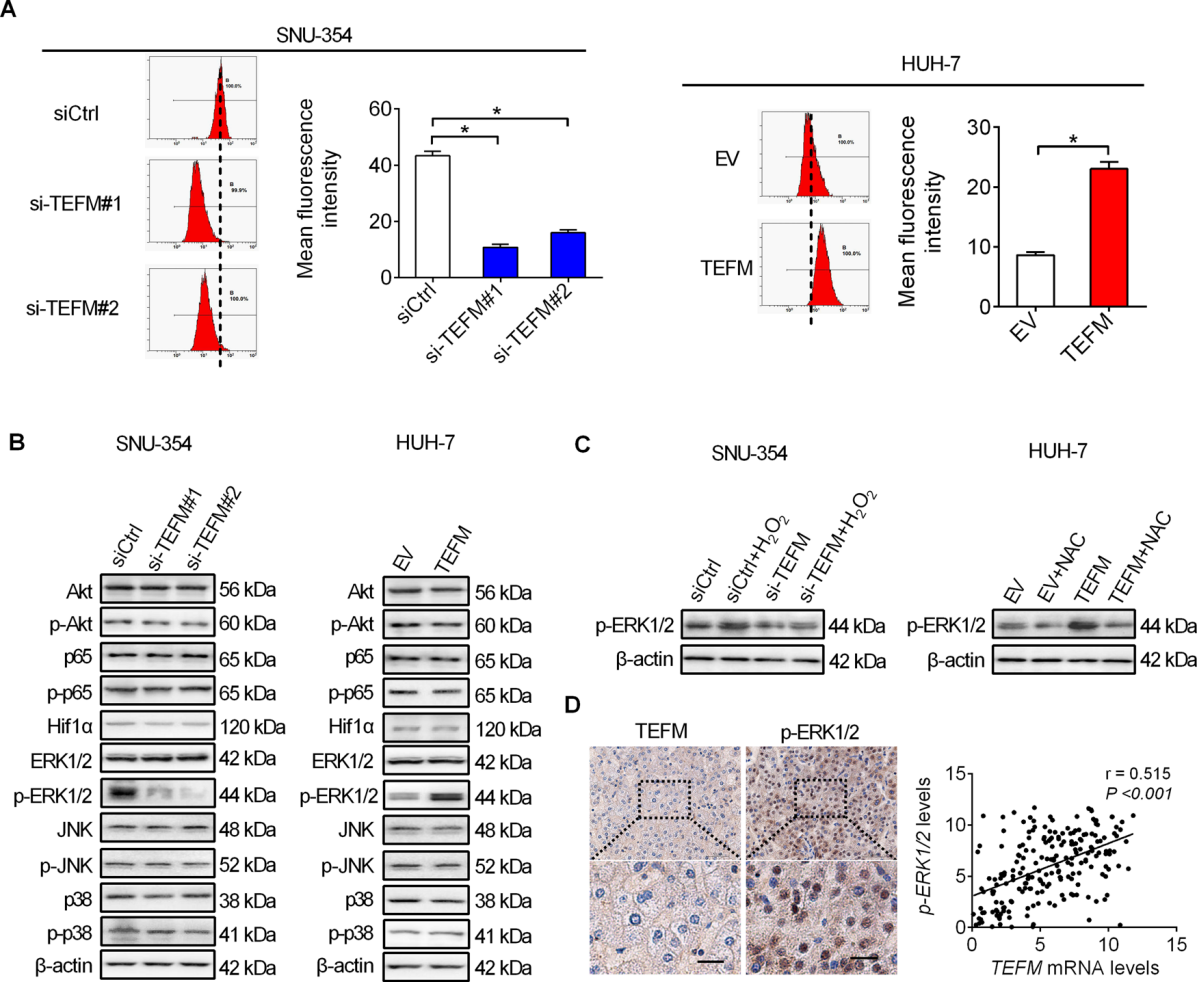
TEFM activated ROS/ERK signaling in HCC cells. **A** Flow cytometry for intracellular ROS level in SNU-354 with TEFM knocked-down and HUH-7 cells with TEFM over-expression. **B** The expression levels of AKT, p-AKT, p65, p-p65, Hif-1α, Erk1/2, p- Erk1/2, JNK, p-JNK, p38, and p-p38 were analyzed by western blot. **C** The expression levels of p-ERK1/2 were analyzed by western blot. Cells were treated with 90 μM H_2_O_2_ or 30 mM NAC for 12 h. **D** Immunohistochemistry analysis for correlation between TEFM and p-ERK1/2 in 209 tumor tissues from HCC patients (scale bars, 20 μm).

We next investigated the molecular mechanisms underlying the oncogenic roles of TEFM in HCC cells. Mitochondria is a major source of ROS, which promotes cancer progression by activating several oncogenic signalings, including AKT, NF-κB, Hif-1α, and MAPK (ERK1/2, JNK, p38)^11^. Accordingly, we firstly analyzed the effect of TEFM on intracellular ROS production in HCC cells. ROS level was significantly decreased upon TEFM knockdown in SNU-354 cells, while increased in HUH-7 cells upon TEFM over-expression (Fig. 7A). In addition, we found that TEFM knockdown suppressed the activation of ERK signaling in SNU-354 cells, as analyzed by western blot. By contrast, TEFM over-expression activated ERK signaling in HUH-7 cells (Fig. 7B). Furthermore, treatment with H_2_O_2_ or NAC (an ROS scavenger) significantly attenuated TEFM knockdown- or over-expression-regulated ERK signaling (Fig. 7C), indicating the activation of ROS/ERK signaling by TEFM in HCC cells. To provide further support, we examined the levels of phosphorylated ERK (p-ERK1/2) in tumor tissues from 209 HCC patients, and found a significant positive correlation between the IHC scores of TEFM and p-ERK1/2 (*r* = 0.515, *p* < 0.001) (Fig. 7D).

**Fig. 8:**
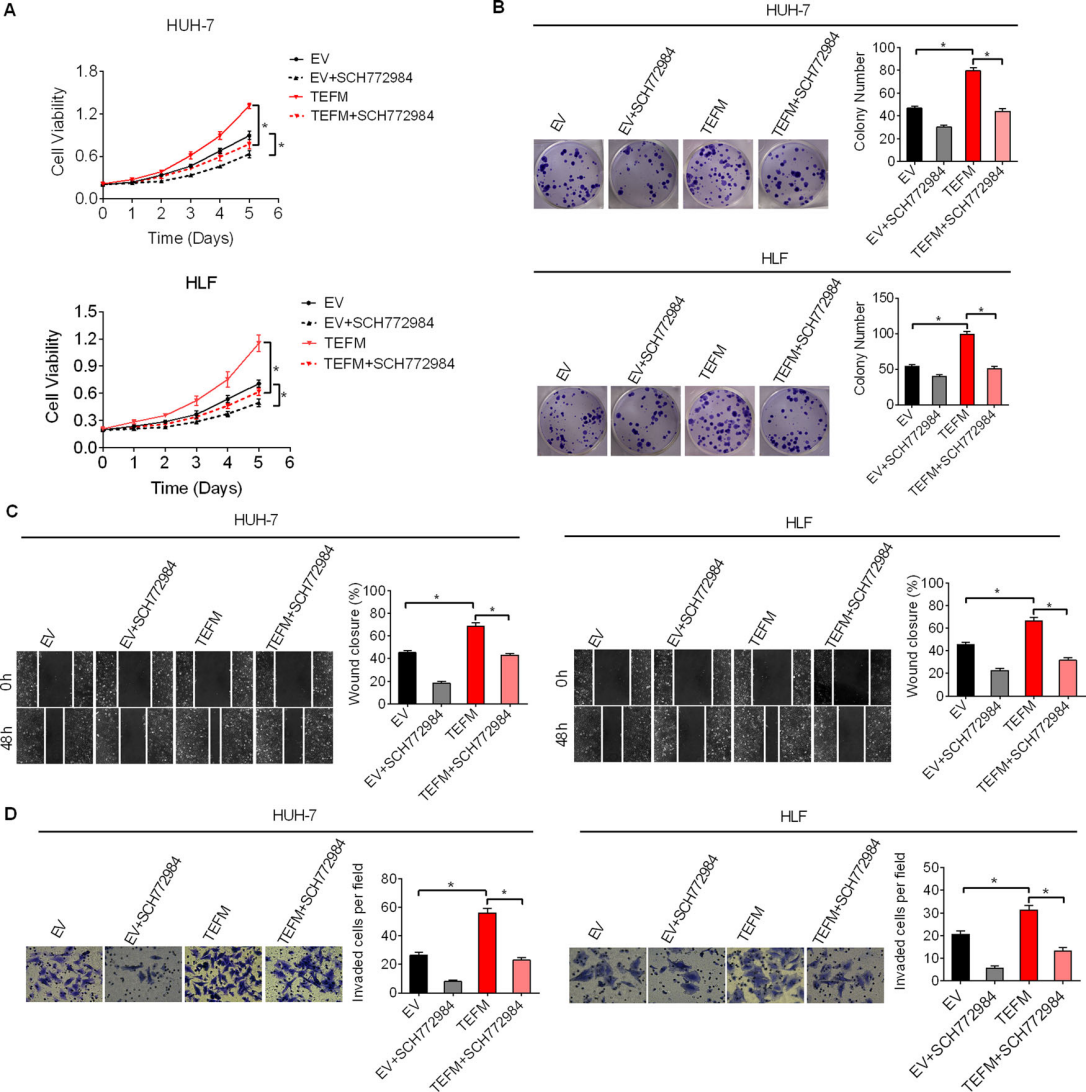
TEFM promoted HCC growth and metastasis through activation of ROS/ERK signaling. **A**, **B** Cell proliferation was analyzed by MTS and colony formation assays in HUH-7 and HLF cells treated with 20 nM SCH772984 or DMSO for 12 h. **C**, **D** Cell metastasis was analyzed by cell migration and invasion assays in HUH-7 and HLF cells treated with 20 nM SCH772984 or DMSO for 12 h.

To further test whether the promotion of HCC growth and metastasis by TEFM is caused by activation of ROS/ERK signaling, we treated HCC cells with a specific ERK inhibitor SCH772984. As shown in Figs. 8A and 7D, SCH772984 treatment clearly suppressed HCC growth and metastasis promoted by TEFM over-expression in HUH-7 and HLF cells. In concordance with the results of inhibition of ERK by SCH772984 treatment, siRNA-mediated knockdown of ERK1/2 also markedly attenuated the growth and metastasis abilities of HUH-7 cells promoted by TEFM over-expression. These findings collectively suggest that TEFM promoted HCC progression through activation of ROS/ERK signaling.

Given that mitochondrial oxidative phosphorylation also plays critical roles in the progression of several tumor types^12^, we therefore further evaluated the effect of TEFM on mitochondrial OCR by using seahorse machine. As shown in Fig. S5, knockdown of TEFM significantly increased the OCR in SNU-354 cells, while TEFM over-expression decreased OCR in HUH-7 cells, suggesting a promotive role for TEFM in mitochondrial oxidative phosphorylation. Accordingly, we cannot exclude the contribution of increased mitochondrial oxidative phosphorylation to TEFM-promoted HCC progression, although our data have demonstrated the involvement of ROS/ERK signaling activation in the oncogenic functions of TEFM in HCC.

## Discussion

In the present study, we observed that TEFM expression is significantly increased in HCC tumor tissues and cell lines mainly due to the down-regulation of miR-194-5p. We showed also that TEFM expression was positively associated with tumor size and vascular invasion, as well as poor patient survival in HCC. In agreement with our findings in HCC, increased expression of another mitochondrial transcription factor TFAM has also been observed in various human cancers, including breast cancer, bladder cancer, colon cancer, glioma, and non-small cell lung cancer^13–17^. Increased TFAM expression has also been shown to predict poor clinical outcome in several types of malignancies, including ovarian cancer, breast cancer, and endometrial carcinoma^13,18,19^. These results collectively indicate that dysregulated mitochondrial transcription plays a critical role in the progression of cancer.

Increased expression suggests an oncogenic role for TEFM in HCC. In this connection, the effects of TEFM on HCC growth and metastasis were analyzed. TEFM knockdown suppressed HCC growth, while its over-expression enhanced HCC growth. In addition, TEFM promoted HCC growth through promoting G1–S cell transition and suppressing cell apoptosis. Similar to the functions of TEFM in HCC, another mitochondrial transcription regulator TFAM also has been reported to induce G1 cell cycle transition, but inhibit cell apoptosis in glioma and non-small cell lung cancers^17,20^. Besides the tumor growth promoting role, increased TFAM also confers resistance to ionizing radiation in several types of malignances^17,21,22^, further supporting an inhibitory role for TFAM in tumor cell apoptosis. Meanwhile, we also tested the effect of TEFM on metastasis of HCC and found that TEFM knockdown decreased the migration and invasion of SNU-354 and SNU-739 cells, while TEFM over-expression enhanced the migration and invasion of HUH-7 and HLF cells. In line with us, a previous study in glioma cells also has shown that increased TFAM promoted tumor cell migration^20^. Furthermore, we showed that TEFM promoted HCC metastasis by inducing EMT, which is consistent with another study of TFAM in the promotion of colorectal cancer (CRC) cells^23^.

MiR-194-5p is a highly conserved miRNA that has multiple targets, and its tumor-suppressive effects have been reported in many different types of cancer, including HCC. For example, miR-194-5p has been reported to inhibit the metastasis of bladder cancer cell by targeting E2F3^24^. In addition, miR-194-5p has also been demonstrated to inhibit nephroblastoma cell metastasis and EMT by targeting Crk^25^. In HCC tissues, significantly down-regulated miR-194-5p was observed compared to precancerous tissues, and its down-regulation contributed to cancer development and progression^26,27^. In our study, bioinformatics analysis also indicated that the down-regulation of miR-194-5p predicts poor survival for patients with HCC. These observations suggest that miR-194-5p functions as a novel tumor suppressor in various tumor types mainly through targeting different oncogenes. Furthermore, we found that miR-194-5p was involved in the over-expression of TEFM and thus its oncogenic functions in the promotion of HCC growth and metastasis. However, we cannot rule out the possibility that other regulators may also lead to TEFM over-expression in HCC, which still needs further investigations.

TEFM is a novel regulator in the transcription of mitochondrial genes, which encodes 13 subunits of the oxidative phosphorylation machinery^28^. Dysfunction of oxidative phosphorylation has been reported to increase mitochondrial ROS production, which plays critical roles in cancer progression by activating several oncogenic signalings^18,29^. Our results revealed that TEFM increased cellular ROS levels and subsequently activation of ERK signaling in HCC cells. In addition, we demonstrated that TEFM exerts its tumor growth and metastasis promoting effects through increasing ROS production and subsequently activation of ERK signaling. However, given that TEFM also promoted mitochondrial oxygen consumption in HCC cells, we therefore cannot exclude the contribution of increased mitochondrial oxidative phosphorylation to TEFM-promoted HCC progression.

Altogether, our study to date indicates that TEFM expression is increased in HCC and its high expression predicts poor prognosis for HCC patients. TEFM plays a critical oncogenic role by promoting both growth and metastasis of HCC. Our study suggests TEFM as a promising prognostic factor and therapeutic target for controlling HCC progression.

## Supplementary information

Supplementary figures and tables.
