# Supplementary material for: Elevated TEFM expression promotes growth and metastasis through activation of ROS/ERK signaling in hepatocellular carcinoma
Source: Cell Death Dis. 2021 Mar 26;12(4):325. doi: 10.1038/s41419-021-03618-7 (PMC7997956; doi:10.1038/s41419-021-03618-7)
Supplement: Supplementary file 1 — Supplementary figures and tables. [file 41419_2021_3618_MOESM1_ESM.docx]

**Supplemental information**

**Elevated TEFM expression promotes growth and metastasis through activation of ROS/ERK signaling in hepatocellular carcinoma**

**Supplemental figures**

**Figure S1.** The prognostic significance of TEFM in HCC was analyzed using the Kaplan–Meier Plotter. **(A)** Overall Survival (OS). **(B)** Progression Free Survival (PFS). **(C)** Disease Free Survival (DFS). **(D)** Disease Free Survival.

**
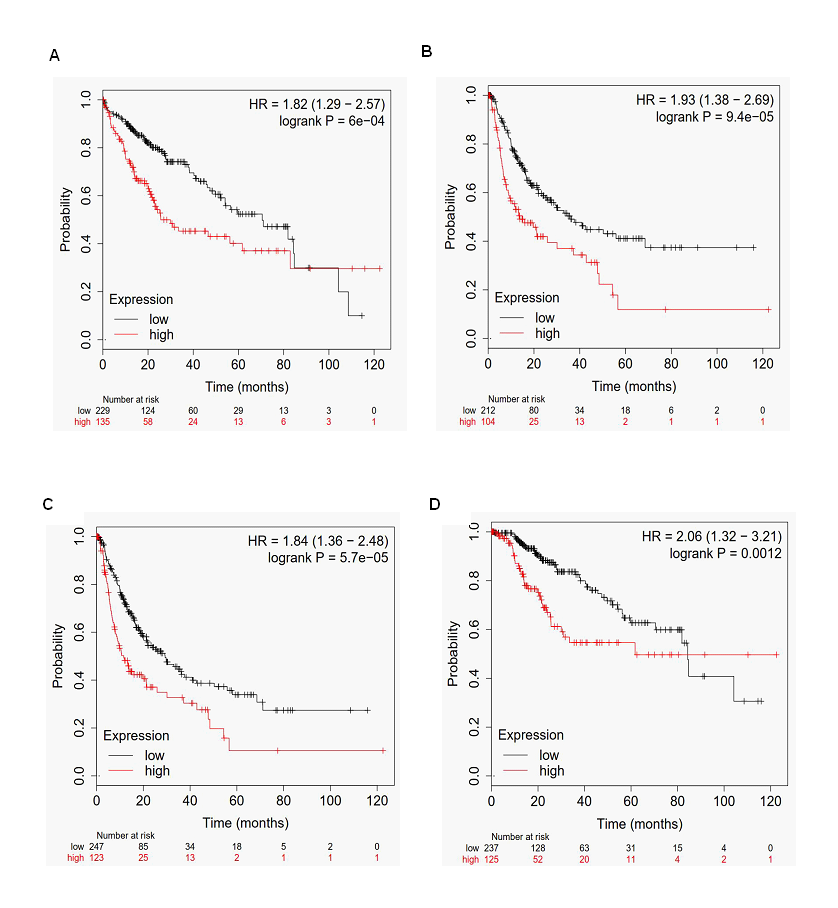
**

**Figure S2. (A and B)** Stable knockdown of TEFM in SNU-354 cells was determined by qRT-PCR and Western blot analysis.

**
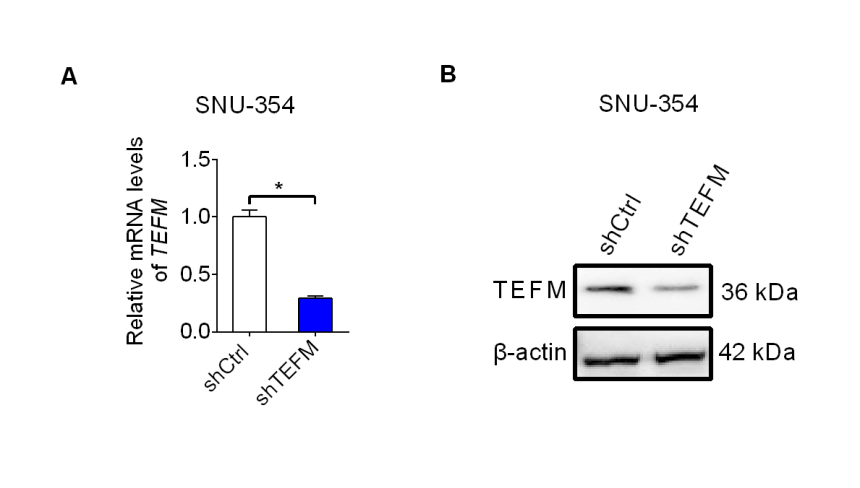
**

**Figure S3. (A)** Top four predicted miRNAs targeting TEFM using the microRNA Data Integration Portal (mirDIP)-based target prediction. **(B)** Bioinformatics analysis based on the ENCORI database[^1^](#_ENREF_1) for the prognostic significance of miR-132-3p in HCC. **(C)** Bioinformatics analysis based on the ENCORI database for the correlation between the expressions of miR-132-3p and SLC25A20 in HCC.

**
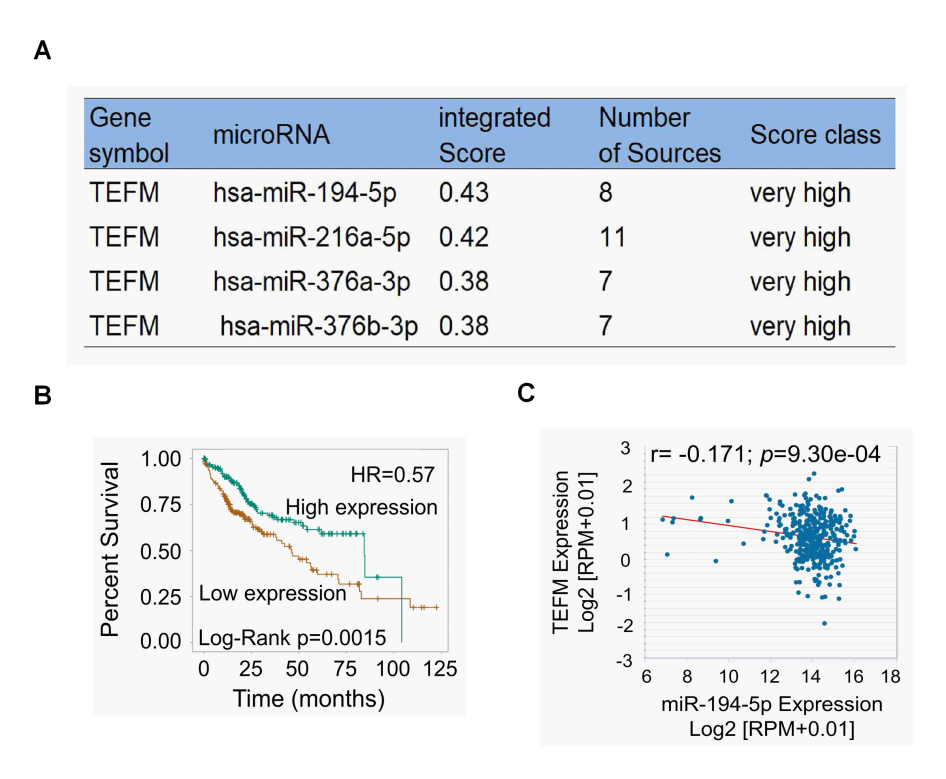
**

**Figure S4. (A)** Total and phosphorylated ERK1/2 expressions were determined by Western blot analysis in HUH-7 cells with indicated treatment. **(B and C)** Cell proliferation was determined by MTS and colony formation assays in HUH-7 cells with indicated treatment. **(D and E)** Cell metastasis was determined by wound healing and matrigel invasion assays in HUH-7 cells with indicated treatment.

**
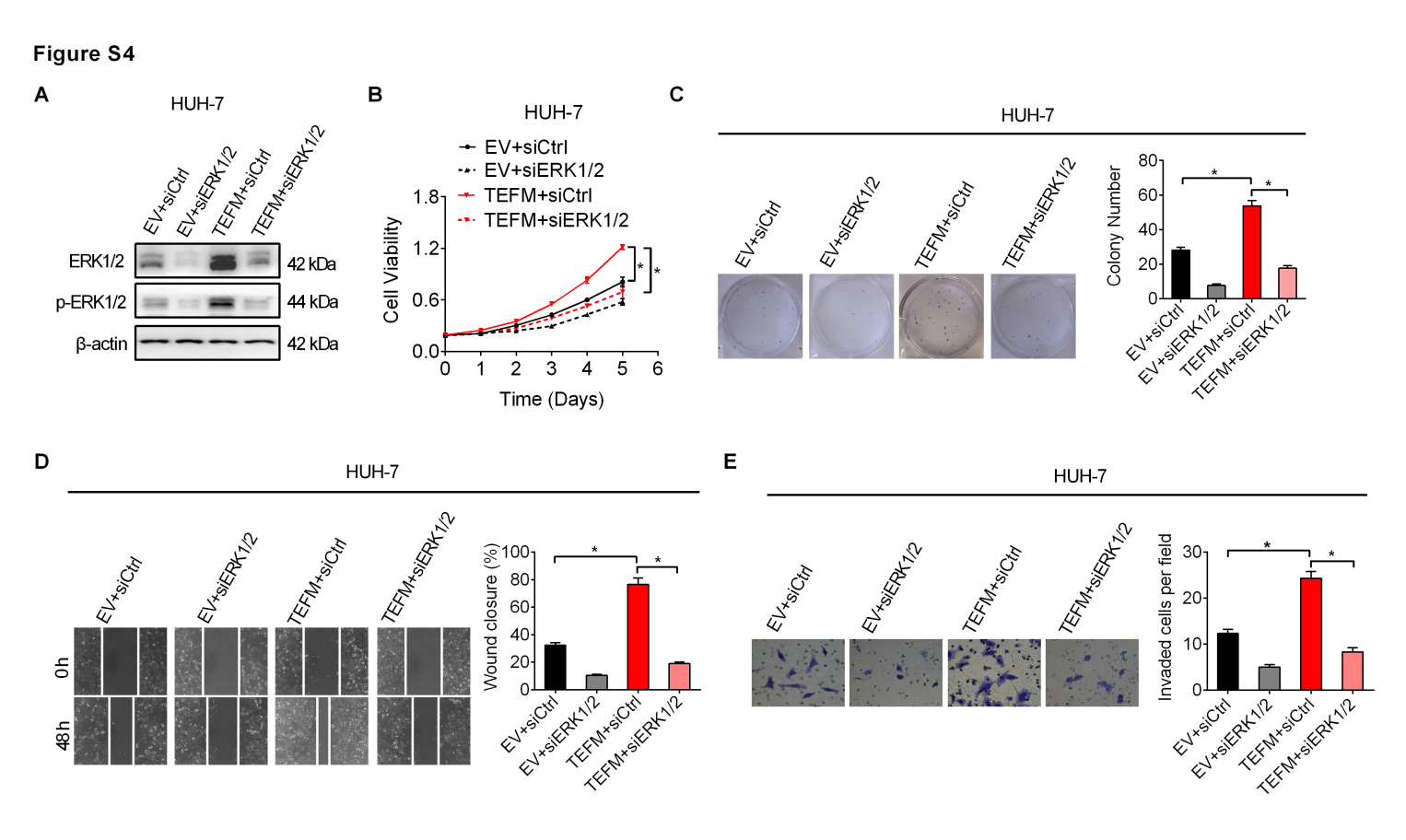
**

**Figure S5.** Oxygen consumption rate (OCR) was evaluated with a seahorse analyzer in SNU-354 and HUH-7 cells when TEFM was knocked-down or over-expressed.

**
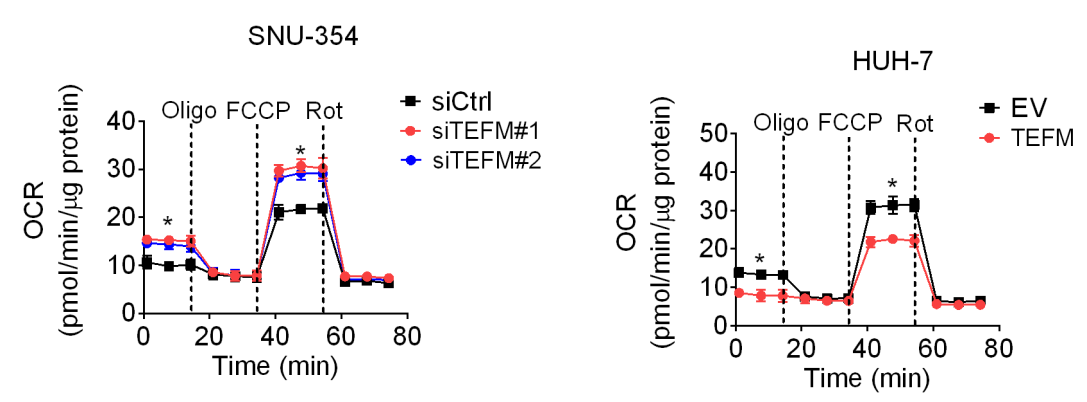
**

**Supplementary Tables**

**Table 1. Correlation between the expression of TEFM and clinicopathologic features in 209 HCC patients.**

| **Variables** | **No. of cases (%)** | **TFB2M expression** | | ***p* value** |
| --- | --- | --- | --- | --- |
|  |  | **Low** | **High** |  |
| All | 209 (100%) | 102 | 107 |  |
| Age |  |  |  |  |
| <55 | 86 (41.1%) | 41 | 45 | 0.88 |
| >=55 | 123 (58.9%) | 61 | 62 |  |
| Gender |  |  |  |  |
| Female | 31 (14.8%) | 14 | 17 | 0.70 |
| Male | 178 (85.2%) | 88 | 90 |  |
| HBV |  |  |  |  |
| Negative | 26 (12.4%) | 12 | 14 | 0.84 |
| Positive | 183 (87.6%) | 90 | 93 |  |
| AFP (ug/ml) |  |  |  |  |
| <200 | 117 (55.9%) | 52 | 65 | 0.17 |
| >=200 | 92 (44.1%) | 50 | 42 |  |
| Maximum diameter of lesion |  |  |  |  |
| <5 | 121 (57.8%) | 67 | 54 | ***0.04*** |
| >=5 | 88 (42.2%) | 35 | 53 |  |
| PVTT |  |  |  |  |
| No | 179 (85.6%) | 94 | 85 | ***0.01*** |
| Yes | 30 (14.4 %) | 8 | 22 |  |
| TNM stage |  |  |  |  |
| I+ II | 164 (79.9 %) | 75 | 89 | 0.09 |
| III+ IV | 45 (20.1%) | 27 | 18 |  |
| Differentiation grade |  |  |  |  |
| I+ II | 68 (32.5%) | 31 | 37 | 0.56 |
| III | 141 (77.5%) | 71 | 70 |  |
| Treatment |  |  |  |  |
| Hepatectomy | 150 (71.7%) | 69 | 81 | 0.22 |
| Hepatectomy+ TACE | 59 (28.3%) | 33 | 26 |  |

**Abbreviations**: AFP, alpha-fetoprotein; PVTT, portal vein tumor thrombosis; TNM, tumor-nodes-metastases; TACE, transcatheter arterial chemoembolization.

**Table 2.** Sequence of primers for qRT-PCR analysis

| **1. Primers used in q-PCR analysis** | | | |
| --- | --- | --- | --- |
| *TEFM* | forward primer | ATGAGCGGGTCTGTCCTCTT |  |
|  | reverse primer | AGTACAGGGATGACCTCGACG |  |
| *E-cadherin* | forward primer | AAAGGCCCATTTCCTAAAAACCT |  |
|  | reverse primer | TGCGTTCTCTATCCAGAGGCT |  |
| *Z0-1* | forward primer | CGACCAGATCCTCAGGGTAA |  |
|  | reverse primer | TCCATAGGGAGATTCCTTCTCA |  |
| *N-cadherin* | forward primer | AGCTCCATTCCGACTTAGACA |  |
|  | reverse primer | CAGCCTGAGCACGAAGAGTG |  |
| *Vimentin* | forward primer | GACGCCATCAACACCGAGTT |  |
|  | reverse primer | CTTTGTCGTTGGTTAGCTGGT |  |
| *β-actin* | forward primer | TCGCCTTTGCGATCCG |  |
|  | reverse primer | ATGATCTGGGTCATCTTCTCG |  |
| miR-194-5p | forward primer | GCGGCGGTGTAACAGCAACTCC |  |
|  | reverse primer | ATCCAGTGCAGGGTCCGAGG |  |
| *U6* | forward primer | GCTTCGGCACATATACTAAAAT |  |
|  | reverse primer | CGCTTCACGAATTTGCGTGTCAT |  |

**Table 3.** Primary antibodies used in the study.

| **Antibody** | **Company (Cat. No.)** | **Working dilutions** |
| --- | --- | --- |
| TEFM | NOVUS (NBP1-82109) | WB:1:1000; IHC:1:200 |
| E-cadherin | abcam (ab1416) | WB: 1/1000 |
| Z0-1 | abcam (ab190085) | WB: 1/1000 |
| N-cadherin | abcam (ab98952) | WB: 1/1000 |
| Vimentin | abcam (ab8978) | WB: 1/1000 |
| Ki-67 | Proteintech (27309-1-AP) | IHC:1/300 |
| Akt | Proteintech (10176-2-AP) | WB: 1/1000 |
| p-Akt | Proteintech (66444-1-IG) | WB: 1/1000 |
| p65 | Proteintech (10745-1-AP) | WB: 1/1000 |
| p-p65 | Abcam (b86299) | WB: 1/800 |
| Hif1α | Abcam (ab92498) | WB: 1/600 |
| ERK1/2 | Proteintech (66192-1-lg) | WB: 1/1000 |
| p-ERK1/2 | Abcam (ab223500) | WB: 1/800 |
| JNK | Proteintech (66210-1-Ig) | WB: 1/1000 |
| p-JNK | cell signaling (#9255) | WB: 1/1000 |
| p38 | Proteintech (66234-1-Ig) | WB: 1/500 |
| p-38 | cell signaling (#4511) | WB: 1/800 |
| β-actin | Proteintech (20536-1-AP) | WB: 1/1000 |

**Reference**

1. Li JH, Liu S, Zhou H, Qu LH, Yang JH. starBase v2.0: decoding miRNA-ceRNA, miRNA-ncRNA and protein-RNA interaction networks from large-scale CLIP-Seq data. *Nucleic acids research* 2014, **42**(Database issue)**:** D92-97.
